# Hypergraph Convolution Network Classification for Hyperspectral and LiDAR Data

**DOI:** 10.3390/s25103092

**Published:** 2025-05-14

**Authors:** Lei Wang, Shiwen Deng

**Affiliations:** 1 College of Geographical Sciences, Harbin Normal University, Harbin 150025, China; wanglei@hljit.edu.cn; 2 College of Surveying and Mapping Engineering, Heilongjiang Institute of Technology, Harbin 150050, China; 3 School of Mathematical Sciences, Harbin Normal University, Harbin 150025, China

**Keywords:** superpixels, hypergraph convolutional networks, hyperedge, hyperspectral image

## Abstract

Conventional remote sensing classification approaches based on single-source data exhibit inherent limitations, driving significant research interest in improved multimodal data fusion techniques. Although deep learning methods based on convolutional neural networks (CNNs), transformers, and graph convolutional networks (GCNs) have demonstrated promising results in fusing complementary multi-source data, existing methodologies demonstrate limited efficacy in capturing the intricate higher-order spatial–spectral dependencies among pixels. To overcome these limitations, we propose HGCN-HL, a novel multimodal deep learning framework that integrates hypergraph convolutional networks (HGCNs) with lightweight CNNs. Specifically, an adaptive weight mechanism is first designed to preliminarily fuse the spectral features of hyperspectral imaging (HSI) and Light Detection and Ranging (LiDAR), enhancing the feature representation ability. Then, superpixel-based dynamic hyperedge construction enables the joint characterization of homogeneous regions across both modalities, significantly boosting large-scale object recognition accuracy. Finally, local detail features are captured through a parallel CNN branch, complementing the global relationship modeling of the HGCN. Comprehensive experiments conducted on three benchmark datasets demonstrate the superior performance of our method compared to existing state-of-the-art approaches. Notably, the proposed framework achieves significant improvements in both training efficiency and inference speed while maintaining competitive accuracy.

## 1. Introduction

Land cover mapping serves as a fundamental tool for analyzing and monitoring human activities and natural environmental processes [1]. Advanced remote sensing image processing techniques enable precise land cover identification and robust classification frameworks [2,3,4]. The rapid evolution of remote sensing and Earth observation technologies has revolutionized the way we collect and interpret data, enabling the acquisition of diverse and high-quality information from a multitude of sensors for practical applications [5,6,7]. Among these, hyperspectral imaging (HSI) has emerged as an indispensable tool for land classification due to its unparalleled ability to capture rich spectral information, which allows for the discrimination of subtle differences in land cover characteristics [8,9,10]. Despite its advantages, HSI-based classification is often constrained by limitations in spatial resolution and susceptibility to atmospheric interference and has difficulties in distinguishing different ground objects that produce similar spectral responses. All these factors may reduce the accuracy of the classification results [11,12]. On the other hand, Light Detection and Ranging (LiDAR) technology affords a complementary vista by supplying highly precise elevation data, which exhibit a relatively lower susceptibility to weather conditions and atmospheric perturbations [6,13,14]. This inherent flexibility and robustness make LiDAR indispensable in scenarios where spectral data alone prove insufficient. The complementary integration of HSI and LiDAR data thus offers a powerful approach to leverage their combined strengths. By merging the rich spectral signatures from HSI with LiDAR’s precise 3D structural information, this multimodal approach effectively mitigates the individual limitations of each technique, substantially improving land classification accuracy [15,16].

In recent years, deep learning-based fusion methods combining HSI and LiDAR data have demonstrated impressive performance due to their robust feature extraction capabilities. Among these approaches, convolutional neural networks (CNNs) and transformer-based methods are the most commonly employed. For instance, Xu et al. [17] developed a dual-tunnel CNN framework to extract spectral–spatial features from both HSI and LiDAR data. Additionally, Wang et al. [18] introduced a multi-scale pyramid fusion framework that leverages spatial–spectral cross-modal attention, enhancing classification through effective multi-scale information learning. Wu et al. [19] proposed a new deep learning framework for multimodal remote sensing data classification, utilizing CNNs as the backbone and incorporating an advanced cross-channel reconstruction module. Traditional CNN-based multimodal fusion classification methods suffer from insufficient contextual awareness. Their limited receptive field design can only extract local features and struggles to model long-range dependencies, resulting in weak global information integration capabilities [20,21]. Recently, transformer networks have been introduced to the multimodal remote sensing domain due to their distinctive and powerful global modeling capabilities, demonstrating remarkable performance. The GLT-Net framework utilizes convolutional operators for local spatial feature extraction while employing transformer architecture to model long-range dependencies. Additionally, it incorporates a hybrid strategy combining multi-scale feature fusion with probabilistic decision fusion to enhance performance [22]. Roy et al. [23] presented a multimodal fusion transformer (MFT) network that features multi-head cross-patch attention and uses LiDAR to initialize the classification token. Yao et al. [24] proposed an innovative multimodal deep learning framework designed for processing remote sensing image patches, which utilizes parallel branches of position-sharing vision transformers (ViTs) enhanced with separable convolutional modules. Despite their successes, these methods often face limitations in the number of parameters, necessitating a large number of labeled samples for optimal training and performance.

Graph-based semi-supervised methods enhance classification accuracy by effectively utilizing information from unlabeled samples [25,26]. For example, Xia et al. [27] applied morphological filters to both LiDAR and hyperspectral data to extract features, which were then fused for classification using semi-supervised graph fusion. Du et al. [28] proposed constructing multimodal graphs for feature fusion, employing graph-based loss functions to guide the feature extraction network. Additionally, Wang et al. [29] introduced a classification method for HSI and LiDAR data based on a dual-coupled CNN-GCN structure. While these graph-based methods have made notable strides in improving classification accuracy by fusing complementary information from two modalities, many existing approaches overlook the complex higher-order inter-modal and intra-modal correlations prevalent in real-world multimodal data. In traditional graph convolutional neural network methods, pairwise connections among data are employed. However, there are limitations in expressing the correlations of multimodal data. The structure of multimodal data extends beyond pairwise connections, and a hypergraph structure can be used for multimodal data modeling. Hypergraphs are capable of encoding high-order data correlations by using their hyperedges without degree constraints [30]. Ma et al. proposed a feature fusion hypergraph neural network for HSI classification. It extracts spatial and spectral features to generate hyperedges for constructing a hypergraph representing HSI features [31]. However, this pixel-wise graph construction method (where each pixel serves as a node and the hyperedge quantity is a multiple of the node count) generates significant computational overhead. EHGNN takes superpixels as the nodes of the hypergraph and uses the KNN algorithm to construct hyperedges for hypergraph feature learning [32]. Xu et al. established a hypergraph model at the superpixel level. This model not only fuses the local homogeneity and complex correlations of HSI but also consumes very little computational resources [33]. Although using superpixels as graph nodes reduces the computational load, it leads to the loss of pixel-level features and easily causes over-smoothing. In this paper, we propose to use pixels as the nodes of the hypergraph, extract superpixels from HSI and LiDAR, respectively, and use the superpixels as hyperedges to construct an adjacency matrix for feature learning. This way, not only can the computational load be greatly reduced but also pixel-level features can be effectively retained without over-smoothing. More importantly, it can naturally depict the homogeneous structures of the two modalities. The main contributions can be summarized as follows:

- In this study, we pioneer the application of HGCNs to the classification tasks of HSI and LiDAR data, enabling the capture of long-range dependencies while simultaneously characterizing the spatial structural properties of both HSI and LiDAR. By integrating HGCNs with a lightweight CNN, our approach effectively extracts local features while fully leveraging the synergistic advantages of both architectures.
- For HSI and LiDAR data, we employ SLIC [34] and Felzenszwalb [35] segmentation methods, respectively. Our innovative strategy of constructing hyperedges using superpixels maximizes the utilization of homogeneous information during feature extraction. This design not only preserves pixel-level discriminative features but also significantly reduces computational overhead.
- Extensive experimental results demonstrate that the proposed HGCN-HL model achieves remarkable performance in HSI and LiDAR classification tasks, outperforming state-of-the-art methods. Benefiting from the inherent advantages of its lightweight architecture, HGCN-HL achieves substantial speed improvements in both the training and testing phases, exhibiting superior computational efficiency compared to other leading networks.

The remainder of this paper is structured as follows. Section 2 introduces the proposed methodology, detailing the framework and key innovations. Section 3 presents the experimental setup, including datasets, implementation details, and comparative results. Section 4 provides an in-depth analysis and discussion of the method’s performance and limitations. Finally, Section 5 concludes this paper with key findings and potential future research directions.

## 2. Method

**Figure 1:**
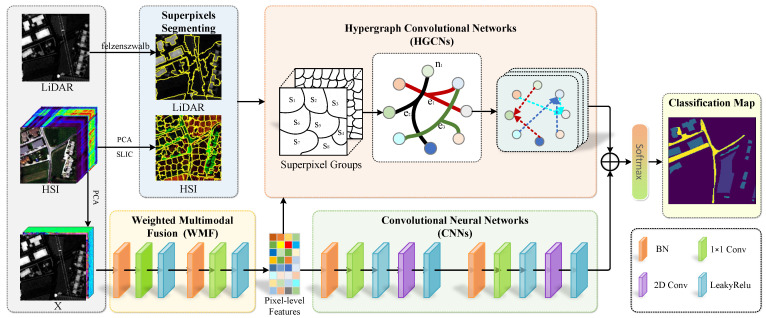
Architecture of the proposed method.

Figure 1 illustrates the framework of the proposed method. We represent the hyperspectral image as $X_{H}\in R^{H\times W\times B}$ and the corresponding LiDAR image as $X_{L}\in R^{H\times W}$, where *H* and *W* are the spatial dimensions and *B* is the number of spectral bands in the hyperspectral image. All pixels are classified into *C* categories, denoted as $C=(y_{1},y_{2},\ldots ,y_{C})$. First, we normalize $X_{H}$ and $X_{L}$ on a channel-wise basis to obtain ${\bar{X}}_{H}$ and ${\bar{X}}_{L}$. Next, we perform principal component analysis (PCA) on the normalized hyperspectral image ${\bar{X}}_{H}$, reducing it to $\bar{B}$ spectral bands, represented as ${\bar{X}}_{H-PCA}\in R^{H\times W\times \bar{B}}$. We then conduct a preliminary fusion of the two modalities along the spectral bands, creating a multimodal dataset defined as $X=\left[{\bar{X}}_{H-PCA},{\bar{X}}_{L}\right]\in R^{H\times W\times (\bar{B}+1)}$, where [·,·] indicates the concatenation operation along the spectral dimension. We provide our source code at https://github.com/giswl/HGCN-HL (accessed on 9 April 2025) to support the remote sensing research community.

### 2.1. Weighted Multimodal Fusion (WMF)

The Weighted Multimodal Fusion (WMF) mechanism is proposed to effectively integrate HSI and LiDAR data through adaptive feature weighting. This approach dynamically assigns importance weights to different modalities based on their discriminative contributions to the classification task. The HSI and LiDAR data are processed using a 1×1 convolution, where $x_{j}^{l}$ denotes the *j*th output feature in the *l*th layer.

(1) $$x_{j}^{l}=f\left(w_{j}^{l}\cdot \mathrm{BN}\left(x^{l-1}\right)+b_{j}^{l}\right),$$

where $w_{j}^{l}$ is the weight parameter of the *l*th layer corresponding to the *j*th output feature. The notation $\mathrm{BN}(x^{l-1})$ indicates that the output $x^{l-1}$ from the (l−1)th layer undergoes a batch normalization operation. Additionally, $b_{j}^{l}$ represents the bias parameter of the *l*th layer corresponding to the *j*th output feature. f(·) is an activation function, such as LeakyReLU(·).

### 2.2. Feature Extraction via CNNs

Convolutional neural networks (CNNs) inherently model inter-feature contextual relationships, enabling robust inference and state-of-the-art performance in HSI and LiDAR classification. Their hierarchical architecture extracts high-level abstract features through localized receptive fields, yielding semantically discriminative representations from complex multimodal data. To extract spatial–spectral features from multimodal data and reduce the model parameters, we employ the spatial–spectral convolution proposed by Liu et al. [36]. The 3D convolution kernel can be decomposed into two simpler convolution kernels: a 1×1 convolution kernel and a 2D convolution kernel. Thus, the spatial–spectral convolution layer can be modeled as

(2) $$x_{p}^{l+1}=\mathrm{LeakyReLU}\left(f_{2D}*\mathrm{LeakyReLU}\left(f_{1\times 1}*\mathrm{BN}\left(x_{p}^{l}\right)\right)\right),$$

where $f_{1\times 1}$ and $f_{2D}$ are the 1×1 and 2D convolutional filters with multiple kernels, BN(·) represents the batch normalization operation, and ‘*’ denotes the convolution operator.

In a conventional standard convolution, a single kernel processes all the channels of the input feature map simultaneously. This implies that the number of parameters per kernel grows proportionally with the input channels, leading to a rapid escalation in both computational cost and parameter count. In this work, the 2D convolutional layer employs depthwise convolution, where each channel of the input feature map is processed by an independent convolutional kernel. Specifically, for an input feature map with $C_{in}$ channels, the depthwise convolution utilizes $C_{in}$ separate kernels, each with a depth of 1. Consequently, each kernel only performs a convolution on a single corresponding input channel, significantly reducing both computational complexity and parameter count.

### 2.3. Multiple Hyperedge Fusion

**Figure 2:**
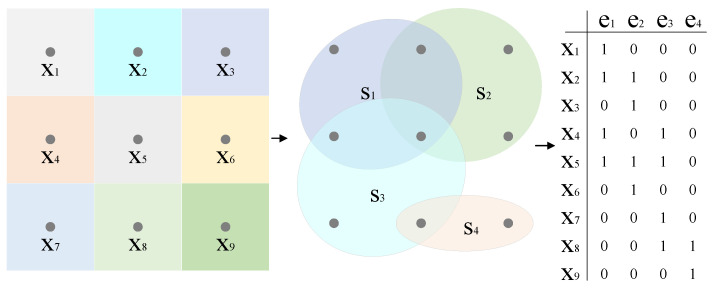
The process of establishing hyperedges.

In conventional GCN-based approaches, graphs are typically constructed and learned using unimodal features. This limitation stems from their reliance on adjacency matrix A as the input, which inherently constrains the number of edges. By contrast, our method employs incidence matrix H to represent hypergraph topology, enabling the simultaneous modeling of both hyperspectral and LiDAR data modalities. This framework requires joint feature generation from the two distinct data sources. As illustrated in Figure 2, we developed a multimodal hypergraph to effectively capture the intrinsic relationships within multimodal data by utilizing superpixels. The Simple Linear Iterative Clustering (SLIC) algorithm, an adaptation of k-means clustering, effectively partitions HSI into homogeneous superpixel regions while preserving local spectral–spatial consistency. After applying SLIC, the HSI is divided into *N* superpixels, represented as $S=s_{1},s_{2},\ldots ,s_{N}$. Each superpixel serves as a hyperedge, which is then flattened with the dataset X, resulting in $X_{flatten}\in R^{HW\times (\bar{B}+1)}$. The relationship between the pixels and superpixels is defined as follows:

(3) $$h\left(x_{i},s_{j}\right)=\left\{\begin{array}{cc} 1, & \mathrm{if}\;x_{i}\in s_{j}\\ 0, & \mathrm{if}\;x_{i}\notin s_{j}, \end{array}\right.$$

where $(h\left(x_{i},s_{j}\right)$) indicates whether pixel $(x_{i}$) belongs to superpixel $\left(s_{j}\right)$. This binary mapping enables the construction of the incidence matrix.

Based on this relationship, an incidence matrix $\left(H\in R^{HW\times N}\right)$ can be constructed. Selecting an appropriate number of superpixels (N) is crucial. An insufficient number of superpixels may cause heterogeneity among the pixels within each superpixel, while an excessive number can elevate computational complexity. For HSI, to appropriately scale for ground objects, we introduce $\left(n_{p}\right)$, the number of pixels per superpixel. This leads to the formula $\left(N=\frac{H\times W}{n_{p}}\right)$, which helps streamline the hyperparameter selection process.

This relationship enables the construction of an incidence matrix $H\in R^{HW\times N}$. Selecting the appropriate number of superpixels (*N*) is essential; too few superpixels can result in heterogeneous pixels within each superpixel, while too many can lead to increased computational complexity. To properly scale for ground objects in HSI, we introduce $n_{p}$, the number of pixels per superpixel, resulting in the expression $N=\frac{H\times W}{n_{p}}$. This method simplifies the hyperparameter selection process in practice. By applying a specific value of $n_{p}$, we generate hyperpixels $S_{H}$ and their corresponding incidence matrices $H_{H}$. For LiDAR data, we leverage the Felzenszwalb algorithm, a well-known and effective segmentation method, to create superpixels $S_{L}$ along with their associated incidence matrices $H_{L}$. The Felzenszwalb algorithm is adept at exploiting the geometric and intensity information in LiDAR data to produce meaningful superpixel segments. The fusion of hyperedges derived from these multimodal features is accomplished by concatenating the respective incidence matrices, as described by the following equation:

(4) $$H_{\mathrm{fuse}}=\left[H_{H},H_{L}\right],$$

where [·,·] represents the concatenation operation. This concatenation operation effectively combines the complementary information from HSI and LiDAR data. By integrating the incidence matrices, we can take advantage of the rich spectral details from HSI and the accurate geometric information from LiDAR, enabling a more comprehensive and robust representation of the scene.

### 2.4. Hypergraph Convolution Neural Network

A hypergraph generalizes traditional graphs by allowing hyperedges to connect arbitrary subsets of vertices. Formally defined as G=(V,E), it consists of vertex set V and hyperedge set E. Unlike graph convolutional networks (GCNs) using adjacency matrices $A\in R^{\left|V|\times |V\right|}$ to model pairwise connections, hypergraphs employ incidence matrices $H\in R^{\left|V|\times |E\right|}$ to capture higher-order relationships. The incidence matrix entries are defined as follows:

(5) $$h\left(v,e\right)=\left\{\begin{array}{cc} 1, & \mathrm{if}\;v\in e\\ 0, & \mathrm{otherwise} \end{array}\right.$$

The hypergraph Laplacian matrix $L\in R^{N\times N}$ is constructed as follows:

(6) $$L=I-D_{v}^{-1/2}HWD_{e}^{-1}H^{⊤}D_{v}^{-1/2},$$

where W is the hyperedge weight matrix, and $D_{v}$, $D_{e}$ are the diagonal matrices encoding the vertex and hyperedge degrees, respectively. Given a vertex v∈V, its degree d(v) is defined as $d\left(v\right)=\sum_{e\in E}\omega \left(e\right)h\left(v,e\right)$. For an edge e∈E, its degree δ(e) is given by $\delta \left(e\right)=\sum_{v\in V}h\left(v,e\right)$. These degree matrices serve to normalize the incidence matrix H, a critical operation for hypergraph analysis.

In the context of HSI and LiDAR fusion, the hypergraph convolution process extends graph convolution principles. For pixel-level feature representation $X_{\mathrm{flatten}}\in R^{HW\times c_{in}}$ and hypergraph association matrix $H_{\mathrm{fuse}}\in R^{HW\times |e|}$, the (l+1)th layer output is computed as follows:

(7) $$X_{\mathrm{flatten}}^{\left(l+1\right)}=\sigma \left(D_{v}^{-1/2}HWD_{e}^{-1}H_{\mathrm{fuse}}^{⊤}D_{v}^{-1/2}X_{\mathrm{flatten}}^{\left(l\right)}\Theta^{\left(l\right)}\right).$$

Here, $W\in R^{\left|e|\times |e\right|}$ is a diagonal weight matrix with trainable parameters $W^{ii}$ representing hyperedge weights. The vertex and hyperedge degree matrices $D_{v}$, $D_{e}$ ensure proper normalization. The learnable parameters $\Theta^{\left(l\right)}\in R^{c_{\mathrm{in}}\times c_{\mathrm{out}}}$ transform input features $X_{\mathrm{flatten}}^{\left(l\right)}$ to output features $X_{\mathrm{flatten}}^{\left(l+1\right)}\in R^{HW\times c_{\mathrm{out}}}$ using the ReLU activation function σ.

### 2.5. Feature Fusion and Classification

Due to the distinct nature of HGCNs and CNNs, the feature distributions from these two branches differ. To effectively integrate these features, this study employs additive fusion, multiplicative fusion, concatenation-based fusion, and attention-based fusion strategies [37], as detailed below:

(8) $$Z_{add}=Z_{G}+Z_{C}$$

(9) $$Z_{mul}=Z_{G}⊙Z_{C}$$

(10) $$Z_{concat}=\left[Z_{G},Z_{C}\right],$$

where $Z_{G}$, $Z_{C}$, and Z represent the outputs of the HGCNs, the CNNs, and the final fused feature map, respectively. [·,·] represents the concatenation operation. The symbol ⊙ denotes the Hadamard product. We use the attention mechanism att($Z_{G}$,$Z_{C}$) to learn their corresponding importance ($\alpha_{g}$,$\alpha_{c}$) as follows:

(11) $$\left(\alpha_{g},\alpha_{c}\right)=att\left(Z_{G},Z_{C}\right).$$

Then, we combine these two embeddings to obtain the final embedding $Z_{att}$ expressed as follows:

(12) $$Z_{att}=\alpha_{g}\cdot Z_{G}+\alpha_{c}\cdot Z_{C}.$$

To train the network, we employ a cross-entropy loss function expressed as follows:

(13) $$L=-\frac{1}{N}\sum_{i=1}^{N}\log\frac{e^{W_{y_{i}}^{T}z_{i}+b_{y_{i}}}}{\sum_{j=1}^{c}e^{W_{j}^{T}z_{i}+b_{j}}}.$$

Here, *N* denotes the number of samples, *c* represents the number of classes, and $z_{i}\in R^{d}$ is the feature representation of the *i*th sample (where *d* is 128 in this paper), belonging to class $y_{i}$. $W_{j}\in R^{d}$ is the *j*th column of the weight matrix $W\in R^{d\times c}$, and $b\in R^{c}$ is the bias term.

## 3. Experiments

This section systematically evaluates the proposed method on three benchmark datasets, with comparative analysis against four state-of-the-art approaches: MFT [23], ExVIT [24], Cross-HL [38], and GAMF [39]. To validate the effectiveness of multi-source data fusion, we designed three experimental configurations: HGCN-HL (HSI) using only hyperspectral image data, HGCN-HL (LiDAR) using only LiDAR data, and HGCN-HL integrating both modalities.

### 3.1. Datasets Description

**Figure 3:**
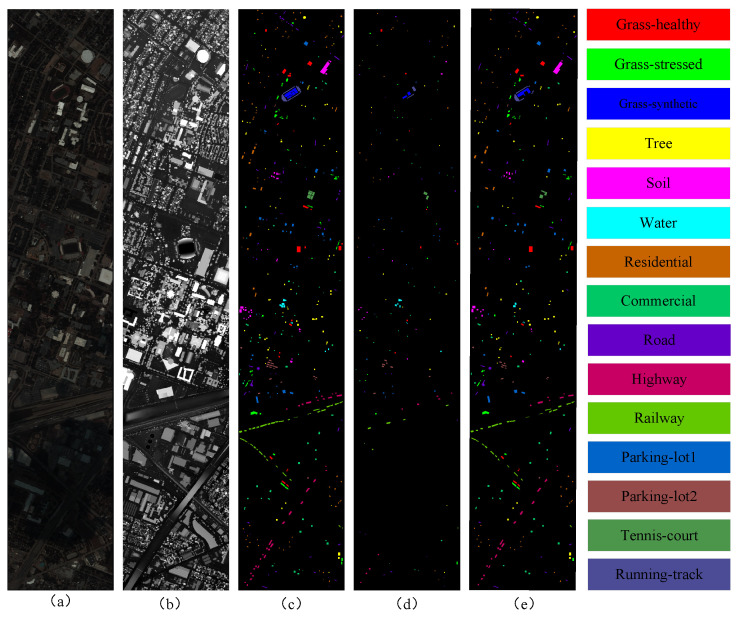
Visualization of Houston 2013 dataset: (**a**) pseudo-color image; (**b**) LiDAR image; (**c**) all samples; (**d**) training samples; and (**e**) test samples.

The Houston 2013 dataset was originally used in the 2013 IEEE GRSS Data Fusion Contest. This dataset includes both hyperspectral and LiDAR images, each with a spatial resolution of 2.5 m and a total image size of 349 × 1905 pixels. The hyperspectral data consist of 144 bands, featuring a spectral resolution of 9.2 nm and a wavelength range from 380 nm to 1050 nm. The dataset is classified into 15 distinct classes. Figure 3 provides the distribution of training and test samples presented as geographical maps. The number of samples used for training and testing in each class is detailed in Table 1. The division of training samples and testing samples is consistent with previous research [40,41].

**Figure 4:**
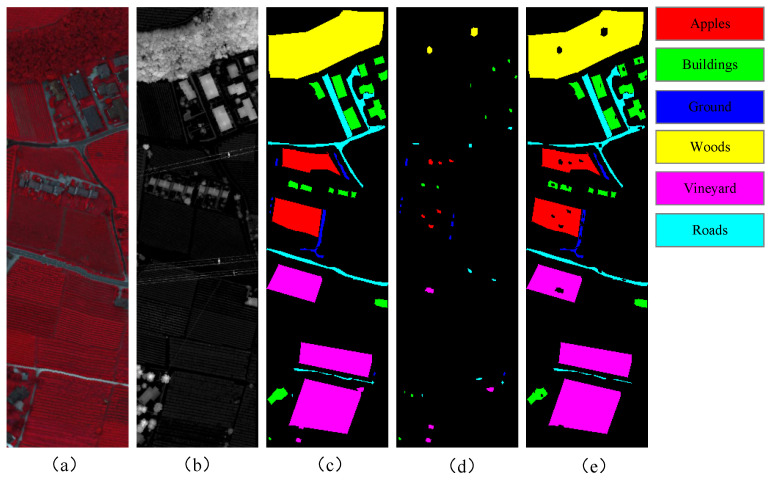
Visualization of Trento dataset: (**a**) pseudo-color image; (**b**) LiDAR image; (**c**) all samples; (**d**) training samples; and (**e**) test samples.

The Trento dataset was collected in the rural areas surrounding the city of Trento, Italy. It includes both HSI and LiDAR data, each with dimensions of 600 × 166 pixels and a spatial resolution of 1 m. The hyperspectral data feature 63 bands, with a spectral range from 420.89 nm to 989.09 nm and a spectral resolution of 9.2 nm. This dataset contains labels for six categories. Figure 4 shows the spatial distribution of the samples. Table 2 presents the stratified sample distribution across all land-cover classes, specifying the precise number of training and testing instances allocated to each category.

**Figure 5:**
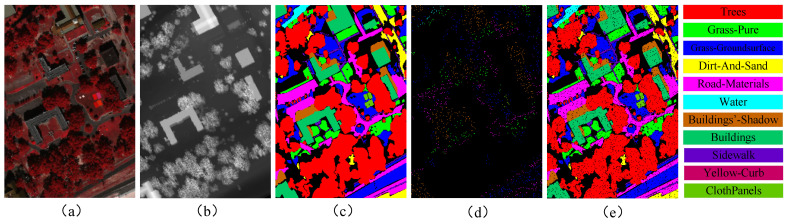
Visualization of MUUFL dataset: (**a**) pseudo-color image; (**b**) LiDAR image; (**c**) all samples; (**d**) training samples; and (**e**) test samples.

The MUUFL Gulfport dataset was acquired in November 2010 through an airborne campaign conducted over the University of Southern Mississippi campus using the Reflective Optics System Imaging Spectrometer (ROSIS) sensor. The HSI component of this dataset comprises a spatial resolution of 325 × 220 pixels with 72 spectral bands across the visible and near-infrared spectrum. Additionally, the dataset includes a LiDAR component containing elevation information represented through two raster layers. After preprocessing, the initial and final eight spectral bands were eliminated due to significant noise contamination, resulting in a refined dataset of 64 spectral bands. The ground truth data encompass 11 distinct urban land-cover classes, totaling 53,687 labeled pixels. The training and test sample sizes for each class are enumerated in Table 3. The visualization of the MUUFL dataset is shown in Figure 5.

### 3.2. Experimental Setting

All experiments in this study were conducted on a uniformly configured high-performance computing platform with the following specifications: equipped with a 13th-generation Intel Core i9-13900K processor (base frequency 3.00 GHz, maximum turbo frequency 5.80 GHz), paired with 64 GB DDR5 memory; the graphics processing unit utilized an NVIDIA GeForce RTX 4090 GPU with 24 GB GDDR6 video memory. The experimental environment operated on Windows 11 Professional OS, primarily developed using Python 3.10.2 and the PyTorch 2.0 framework.

To address the memory overflow issues caused by pixel-level large adjacency matrices in high-resolution image processing, this study adopted the efficient information aggregation strategy from the PyTorch Geometric (PyG) framework to replace traditional adjacency matrix storage methods. The Adam optimizer was selected with key hyperparameters configured as follows: an initial learning rate of 0.0001, and a weight decay coefficient of 0.00001. The maximum training epochs were set to 1000. For comparative methods MFT and Cross-HL, following their original literature settings, the training epochs were uniformly adjusted to 500 to ensure classification accuracy, while all other parameters maintained the recommended configurations from their respective original papers.

### 3.3. Comparison and Analysis of Classification Performance

Three publicly available datasets and four deep learning methods were adopted for performance benchmarking. The proposed approach was rigorously evaluated on HSI-only, LiDAR-only, and an HSI with LiDAR fusion. All experiments were repeated 10 times, with the mean and standard deviation of each metric reported as the final evaluation criteria. Classification results for Houston 2013, Trento, and MUUFL datasets are systematically presented in Table 4, Table 5 and Table 6, respectively. Quantitative metrics including the Overall Accuracy (OA), Average Accuracy (AA), Kappa coefficient (κ), and class-specific accuracy rates are reported.

The proposed HGCN-HL achieved state-of-the-art performance with an OA of 91.20%, AA of 91.71%, and κ of 90.44%, surpassing the suboptimal EXViT approach by a significant margin (>1%) across all evaluation metrics in Table 4. Notably, this represents more than a 6% OA improvement over the GAMF approach that employs simplified feature mapping, demonstrating the efficacy of hypergraph-based modeling for capturing higher-order pixel relationships. Using single-modality data, the OA achieved by HSI and LiDAR are 89.03% and 65.96%, respectively. This indicates that the classification accuracy of LiDAR for this data is relatively low, mainly due to the complexity of the scene objects. However, our method, which utilizes the complementary information of HSI and LiDAR, has achieved the highest accuracy with an OA of 91.20%. Moreover, the classification performance of classes 8, 9, 10, and 11 has significantly improved compared to using only HSI data. Coincidentally, these classes are Commercial Area, Roads, Highways, and Railways, respectively. These classes use relatively similar materials, and our method further improves the classification accuracy by extracting complementary information.

Table 5 presents a comprehensive comparison of classification performance between the proposed HGCN-HL network and benchmark methods on the Trento dataset. The proposed method demonstrates superior performance, achieving higher accuracy in 4 out of 6 classes compared to existing approaches. Notably, our HGCN-HL attains an OA of 99.08%, representing the only method to surpass the 99% threshold and outperforming other state-of-the-art methods by a significant margin. Further analysis reveals that the HGCN-HL using LiDAR-only data achieves an OA of 89.20% on Trento, which shows substantial improvement compared to its performance on Houston 2013 (65.96%) and MUUFL (80.56%). This 23.24% and 8.64% enhancement, respectively, suggests that the LiDAR data in Trento contain more discriminative information, contributing to a 2% Overall Accuracy improvement in the fused model.

Table 6 demonstrates that the proposed HGCN-HL method achieves state-of-the-art performance across multiple categories. Specifically, the method attains the highest classification accuracy in six categories. HGCN-HL achieves the highest OA of 95.42%, outperforming all other methods. In terms of AA, ExViT obtains the highest score (84.50%), while HGCN-HL performs slightly lower (84.05%).

### 3.4. Visual Comparison

**Figure 6:**
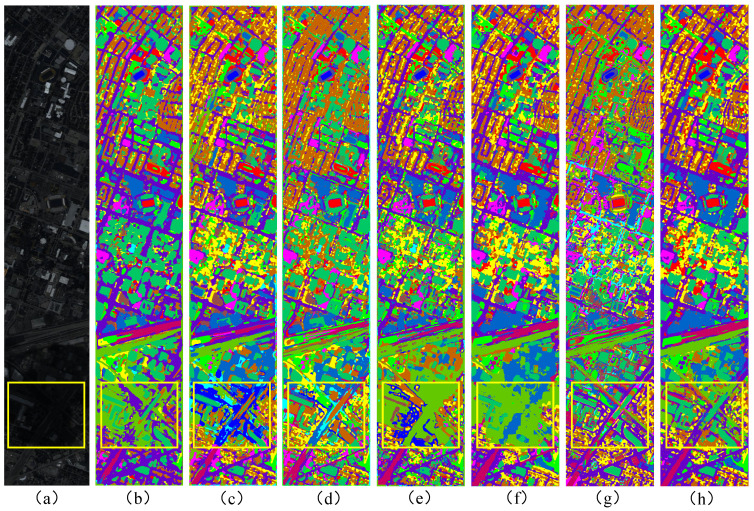
Classification maps of different methods for the Houston 2013 dataset. (**a**) pseudo-color image. (**b**) MFT. (**c**) ExVIT. (**d**) Cross-HL. (**e**) GAMF. (**f**) HGCN-HL (HSI). (**g**) HGCN-HL (LiDAR). (**h**) HGCN-HL.

As illustrated in Figure 6, the classification maps produced by our method exhibit smoother and more continuous feature shapes compared to those generated by other methods, particularly in road areas, where our results are both more complete and geometrically consistent. Notably, our method demonstrates a substantial improvement in accuracy for class nine (Roads) compared to all other methods in Table 4. It is important to note that the yellow-bordered region in the figure is affected by cloudy weather conditions in the original data, resulting in poor classification outcomes for all methods in this area. However, our method demonstrates the best performance by producing smoother and more defined edges around the features. Figure 6f,g show the classification maps derived from HSI and LiDAR data, respectively. Upon examination, it is evident that due to inherent limitations in the characteristics of the data, neither modality alone produces the optimal classification map. In contrast, our method effectively combines the strengths of both data types, resulting in superior classification performance.

**Figure 7:**
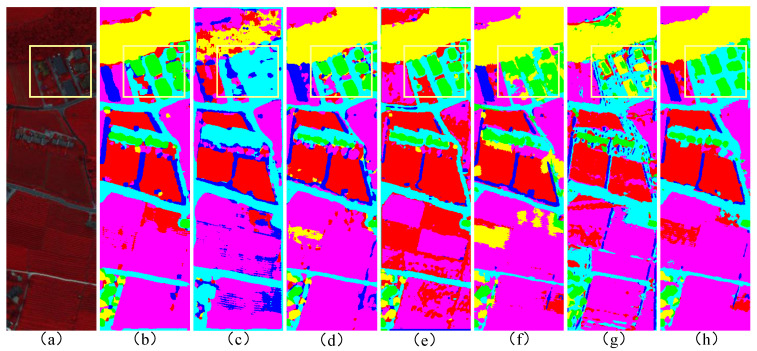
Classification maps of different methods for the Trento dataset. (**a**) pseudo-color image. (**b**) MFT. (**c**) ExVIT. (**d**) Cross-HL. (**e**) GAMF. (**f**) HGCN-HL (HSI). (**g**) HGCN-HL (LiDAR). (**h**) HGCN-HL.

As clearly illustrated in Figure 7, the classification map generated by our HGCN-HL model demonstrates remarkable smoothness characteristics. For large-scale objects within extensive homogeneous regions, the model achieves exceptionally accurate classification results. This superior performance stems from its effective utilization of homogeneous information, which robustly suppresses outlier issues induced by discrete noise in HSI, thereby significantly improving classification accuracy for large-scale features. In contrast, other classification methods exhibit varying degrees of noise interference, leading to misclassification errors that our HGCN-HL model successfully avoids when processing large-scale objects. Particularly noteworthy is the model’s outstanding performance in classifying small objects within the yellow-bordered regions, where HGCN-HL demonstrates substantially superior results compared to other approaches. These small objects are precisely delineated with clear category boundaries, effectively preventing misclassification caused by their complex spectral characteristics.

**Figure 8:**
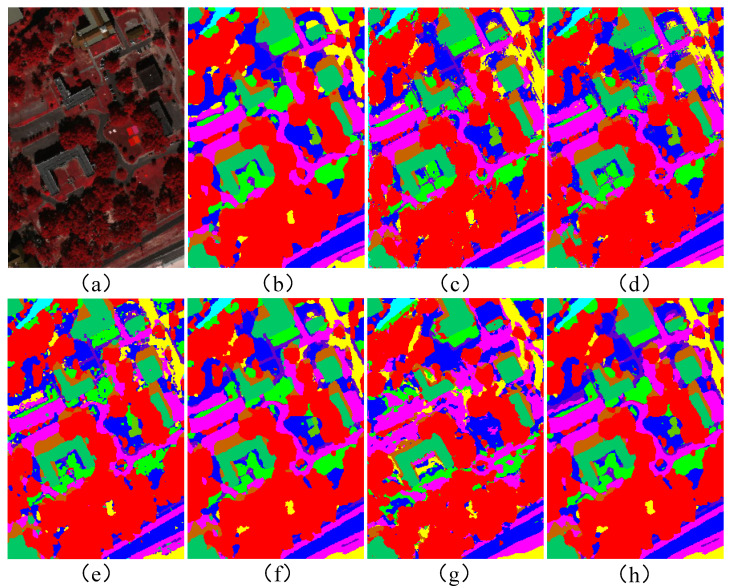
Classification maps of different methods for the Trento dataset. (**a**) pseudo-color image. (**b**) MFT. (**c**) ExVIT. (**d**) Cross-HL. (**e**) GAMF. (**f**) HGCN-HL (HSI). (**g**) HGCN-HL (LiDAR). (**h**) HGCN-HL.

A comparative analysis of Figure 8 reveals that our HGCN-HL model excels in boundary processing compared to alternative methods. The generated classification map displays exceptionally smooth transitions at object boundaries without abrupt artifacts. Moreover, within homogeneous regions, the model completely avoids salt-and-pepper noise-induced misclassification, maintaining consistently high classification accuracy. These advantages establish HGCN-HL as a more reliable solution for HSI and LiDAR data classification tasks.

## 4. Discussion

### 4.1. Analysis of Hyperparamaters

**Figure 9:**
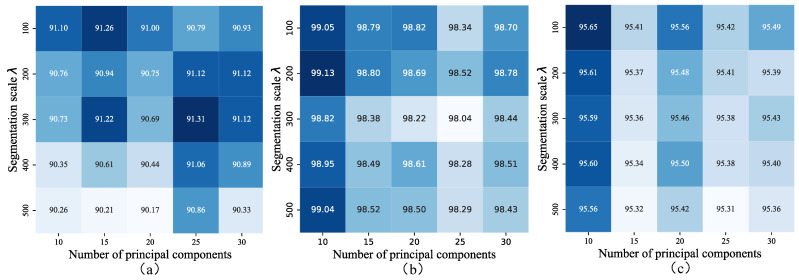
Classification accuracies of HGCN-HL with different hyperparamaters on each dataset. (**a**) Houston 2013. (**b**) Trento. (**c**) MUUFL.

To determine the optimal segmentation scale (λ) and number of principal components (*p*) across different datasets, this study employs a grid search strategy to optimize these two critical hyperparameters in the deep learning model. The search ranges are defined as λ∈{100,200,300,400,500} and p∈{10,15,20,25,30}, with Overall Accuracy (OA) serving as the evaluation metric (see Figure 9). The highest OA values were achieved for the parameter pairs (λ, *p*) = (300, 25), (200, 10), and (100, 10) for the three datasets, respectively. The segmentation scale (λ) governs the size of the constructed graph. Larger values of λ yield fewer hyperedges, where each hyperedge connects pixels across broader ground object regions, thereby enhancing noise suppression. Conversely, smaller segmentation scales produce a higher number of hyperedges but introduce greater noise sensitivity. The grid search revealed that smaller λ and *p* values generally reduce computational overhead, as they decrease the graph size and feature dimensionality.

### 4.2. The Effect of the Different Fusion Methods

As summarized in Table 7, this study systematically evaluates four fusion strategies additive-based (Add), multiplicative-based (Mul), concatenation-based (Concat), and attention-based (Attention) fusion across three benchmark datasets. Experimental results reveal distinct performance variations among the strategies. Specifically, concatenation-based fusion achieved the highest Average Accuracy (AA) of 85.52% ± 0.48 on the MUUFL dataset. This outcome underscores the importance of feature dimension expansion for enhancing discriminative power. Cross-dataset comparisons further demonstrate that optimal fusion strategy selection is dataset-dependent, necessitating alignment with specific data characteristics. Notably, additive-based fusion exhibits the most consistent performance across all three datasets, suggesting its robustness to diverse spectral and spatial feature distributions.

### 4.3. Sample Size Impact on Few-Shot Learning Performance

As comprehensively evaluated in Table 8, Table 9 and Table 10, the proposed HGCN-HL demonstrates consistent superiority over four benchmark methods (MFT, Cross-HL, EXIT, and GAMF) in few-shot hyperspectral classification tasks with 3–9 training samples per class. Under the extreme three-sample condition, HGCN-HL achieves statistically significant OA improvements of 2.78–7.28% compared to suboptimal approaches, attributable to its dual innovation framework. Superpixel-driven homogeneous region segmentation that aggregates spectrally consistent pixels to counteract sample sparsity. Hierarchical hypergraph convolutional networks synergistically model local spatial patterns and global contextual dependencies through multimodal hyperedges. This architecture effectively resolves the information fragmentation problem by establishing robust topological connections between isolated features while simultaneously suppressing noise through adaptive neighborhood relationship weighting. The empirical results validate HGCN-HL’s capability to balance feature granularity and relational reasoning in data-scarce scenarios.

### 4.4. The Effect of Adding Gaussian Noise to HSIs

**Figure 10:**
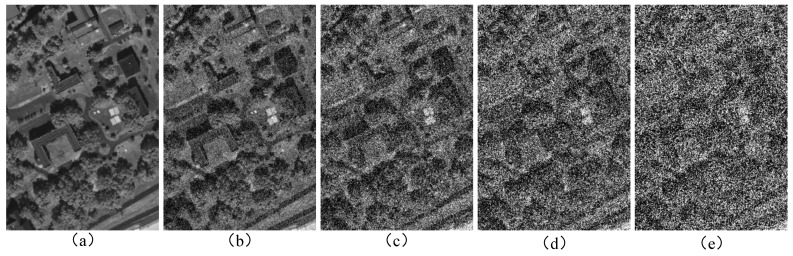
Visualization of the injection of Gaussian noise at various variance levels into the 40th band of the MUUFL. (**a**) Original image. (**b**) δ=0.1. (**c**) δ=0.2. (**d**) δ=0.3. (**e**) δ=0.4.

**Figure 11:**
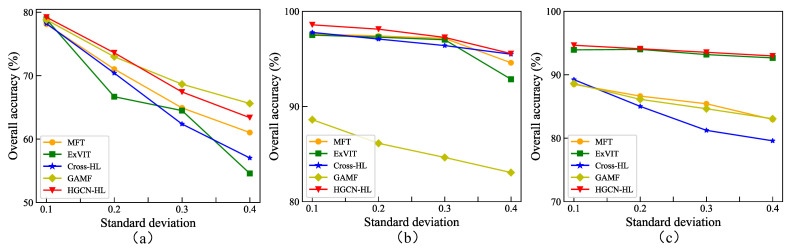
Classification accuracies of different methods for the HSI with the percentage of noise added. (**a**) Houston 2013. (**b**) Trento. (**c**) MUUFL.

To investigate the performance of HGCN-HL under noise interference, we conducted noise testing experiments on all models. In these experiments, we added Gaussian noise with a zero mean and standard deviations of 0.1, 0.2, 0.3, and 0.4 to the original hyperspectral images. Figure 10 shows the visualization results of noise-added images on the MUUFL dataset. As shown in Figure 11, we systematically evaluated the noise resistance of five methods under noise standard deviations ranging from 0.1 to 0.4. In the Houston 2013 dataset, all methods exhibited rapid accuracy degradation with increasing noise levels. While HGCN-HL showed slightly lower accuracy than GAMF at noise levels of 0.3 and 0.4, it achieved significantly higher accuracy than other methods on the Trento and MUUFL datasets, demonstrating superior noise robustness. The noise had a limited impact on graph structure construction. Hypergraph convolution enhances robustness by aggregating neighborhood information within hyperedges to update node representations and reduce noise effects. Furthermore, graph convolution improves noise resistance by leveraging topological information in graph structures to better capture global data relationships.

### 4.5. Ablation Analysis

To rigorously evaluate the contribution of each module, we conducted comprehensive ablation studies on each dataset independently. Our experimental analysis reveals that the synergistic combination of the WMF module, CNN branch, and HGCN branch achieves superior performance compared to their individual implementations, demonstrating their strong complementary characteristics. Specifically, the HGCN branch delivers performance improvements of 0.68%, 4.29%, and 0.4% across the three datasets, respectively in Table 11.

The most remarkable enhancement occurs on the Trento dataset, where the HGCN branch achieves particularly significant gains. This can be attributed to two key factors: (1) The dataset contains numerous large-scale objects that are precisely captured by the hypergraph structure constructed from superpixels in the HGCNs branch. (2) The hypergraph architecture effectively leverages structural information during feature extraction and classification, substantially boosting classification accuracy. In contrast, the CNN branch demonstrates exceptional performance on the Houston 2013 dataset, achieving notable performance gains. Our in-depth analysis indicates that this stems from the dataset’s characteristic small yet complex features, which align perfectly with the strength of CNNs in extracting and processing local patterns. Furthermore, the WMF module contributes substantially to the overall performance through its dual-function 1 × 1 convolutional operation enabling an optimal weighted fusion of HSI and LiDAR data to combine their complementary advantages, and effectively suppressing noise interference in the data.

### 4.6. Comparison of Running Time

Table 12 presents the training and testing times of different methods on each dataset. It should be noted that the time spent on superpixel segmentation and hypergraph construction is incorporated into both training and testing time to ensure fairness. The test pixels in the classified HSI and LiDAR are taken as the testing time. Different from other deep learning methods that use local patches of HSI and LiDAR as inputs, HGCN-HL uses the entire HSI as the input, enabling parallel computation. Patch-based methods perform repetitive feature extraction in overlapping regions, resulting in an increase in computation time. Therefore, HGCN-HL has significantly improved training and testing speeds on the three datasets. The training speed improvement is more pronounced on Trento and MUUFL compared to Houston 2013, mainly due to the large input size and small number of training samples on Houston 2013. However, the testing time on the Houston 2013 dataset is not optimal; this is because HGCN-HL performs feature extraction on the entire image, while other methods only conduct inference on test samples. In practical applications where inference is performed on the entire image, HGCN-HL will demonstrate a significant advantage. In addition, owing to its lightweight network structure, the testing speed of the proposed method has been greatly enhanced, making it more valuable for future practical applications.

## 5. Conclusions

In this paper, the HGCN-HL is proposed for improving the performance of HSI and LiDAR classification by integrating the HGCNs and CNNs subnetworks to effectively capture the complementary spatial and spectral information inherent in HSI and LiDAR datasets. The WMF module achieves preliminary information fusion between HSI and LiDAR while effectively reducing noise. In the HGCN subnetwork, the proposed HGCN model constructs hyperedges using superpixels, leveraging homogeneous information between HSI and LiDAR pixels to naturally realize multimodal data fusion while significantly reducing computational complexity. The CNN subnetwork employs two simple convolutional kernels to minimize computational overhead while effectively extracting heterogeneous information from local spatial domains. Experimental results on three benchmark datasets demonstrate that HGCN-HL achieves outstanding performance. Notably, our method exhibits significant speed advantages during both the training and testing phases. For future work, the existing framework could be extended to investigate alternative hyperedge construction strategies for capturing additional structural information. We will explore the broader applicability of HGCN-HL in fields such as disaster monitoring, precision agriculture, and urban studies.

## Figures and Tables

**Table 1:** List of training samples, test samples, and total samples for each category in the Houston 2013 dataset.

No.	Category	Training Samples	Test Samples	Total Samples
1	Healthy Grass	126	1125	1251
2	Pressed Grass	126	1128	1254
3	Synthetic Grass	70	627	697
4	Trees	125	1119	1244
5	Soil	125	1117	1242
6	Water	33	292	325
7	Residential Area	127	1141	1268
8	Commercial Area	125	1119	1244
9	Roads	126	1126	1252
10	Highways	123	1104	1227
11	Railways	124	1111	1235
12	Parking Lot 1	124	1109	1233
13	Parking Lot 2	47	422	469
14	Tennis Court	43	385	428
15	Running Track	66	594	660
	Total	1510	13,519	15,029

**Table 2:** List of training samples, test samples, and total samples for each category in the Trento dataset.

No.	Category	Training Samples	Test Samples	Total Samples
1	Apple Trees	129	3905	4034
2	Buildings	125	2778	2903
3	Ground	105	374	479
4	Trees	154	8969	9123
5	Vineyards	184	10,317	10,501
6	Roads	122	3052	3174
	Total	819	29,395	30,214

**Table 3:** List of training samples, test samples, and total samples for each category in the MUUFL dataset.

No.	Category	Training Samples	Test Samples	Total Samples
1	Grass-Pure	214	4056	4270
2	Dirt-And-Sand	91	1735	1826
3	Water	23	443	466
4	Buildings	312	5928	6240
5	Yellow-Curb	9	174	183
6	Trees	1162	22,084	23,246
7	Grass-Groundsurface	344	6538	6882
8	Road-Materials	334	6353	6687
9	Buildings’-Shadow	112	2121	2233
10	Sidewalk	69	1316	1385
11	ClothPanels	13	256	269
	Total	2683	51,004	53,687

**Table 4:** Individual class, OA, AA, and Kappa of all methods on the Houston 2013 dataset.

Class	MFT	ExVIT	Cross-HL	GAMF	HGCN-HL (HSI)	HGCN-HL (LiDAR)	HGCN-HL
1	82.30±0.36	83.06±1.86	82.98±0.11	83.05±0.11	83.09±0.03	49.41±10.85	83.08±0.06
2	86.32±3.95	85.72±3.78	86.02±3.69	84.33±1.27	95.36±5.27	48.92±7.46	93.01±6.70
3	98.46±1.13	97.43±2.08	99.29±0.49	95.58±2.33	99.58±0.11	71.49±7.17	99.74±0.15
4	98.39±0.88	93.6±3.17	98.99±1.85	96.04±2.92	90.94±3.04	71.62±4.42	97.05±3.07
5	99.68±0.30	99.83±0.19	99.93±0.11	99.93±0.07	99.98±0.06	81.84±5.36	100.00±0.00
6	93.92±4.65	97.83±2.05	96.22±1.93	92.87±4.26	97.55±2.01	75.87±6.58	98.60±1.85
7	83.88±1.94	92.58±4.70	87.21±2.24	88.48±2.81	85.54±3.27	85.92±2.61	85.90±5.08
8	82.16±4.44	95.23±1.65	77.56±2.25	69.17±5.76	79.34±1.94	84.60±3.37	85.66±5.89
9	89.32±2.74	86.88±2.84	85.36±2.94	71.43±5.27	91.32±2.92	59.23±4.09	96.01±2.17
10	56.84±2.07	67.49±3.80	56.71±5.20	64.90±3.20	63.56±3.32	49.23±5.56	70.72±9.59
11	98.33±1.18	89.85±5.05	93.05±6.75	87.08±6.04	91.56±7.13	87.14±2.92	95.74±4.37
12	93.99±1.92	92.62±2.30	90.43±2.98	90.80±3.87	98.52±1.43	38.65±7.31	98.13±1.36
13	87.68±4.15	88.35±3.48	91.19±2.83	79.12±10.55	78.04±4.32	64.88±2.63	78.63±4.55
14	99.80±0.37	97.09±3.00	99.07±1.10	100±0.00	100.00±0.00	96.56±5.62	100.00±0.00
15	94.61±6.06	98.39±2.59	99.32±0.97	93.62±4.96	99.96±0.08	46.77±9.10	100.00±0.00
OA (%)	88.27±0.28	89.73±0.96	87.47±0.65	84.78±0.43	89.03±0.72	65.96±1.34	91.31±1.54
AA (%)	89.71±0.35	91.06±0.99	89.56±0.5	86.43±0.72	90.29±0.66	67.47±0.88	92.15±1.29
KPP (×100)	87.26±0.31	89.00±1.00	86.42±0.69	83.48±0.47	88.08±0.79	63.11±1.44	90.56±1.68

Bold values indicate the highest performance in each category.

**Table 5:** Individual class, OA, AA, and Kappa of all methods on the Trento dataset.

Class	MFT	ExVIT	Cross-HL	GAMF	HGCN-HL (HSI)	HGCN-HL (LiDAR)	HGCN-HL
1	97.60±0.30	98.02±0.86	99.53±0.42	95.85±0.86	99.56±0.18	99.13±1.73	99.94±0.07
2	98.11±2.92	98.93±0.81	97.49±0.86	99.33±0.35	98.58±0.50	57.78±6.21	99.20±0.35
3	79.44±10.28	89.52±5.04	90.13±7.16	95.03±2.25	98.74±1.40	51.15±5.73	98.18±1.50
4	99.84±0.17	99.99±0.01	99.85±0.15	99.79±0.22	99.61±0.28	93.91±1.07	100.00±0.00
5	99.84±0.24	99.88±0.11	99.98±0.04	99.91±0.05	99.50±0.39	92.76±1.81	99.82±0.30
6	86.91±3.63	92.14±3.49	89.39±2.44	90.92±1.24	72.80±3.01	83.89±5.07	93.40±0.74
OA%	97.78±0.30	98.64±0.40	98.42±0.19	98.28±0.16	96.67±0.37	89.20±0.41	99.13±0.14
AA%	93.62±1.75	96.41±1.16	96.06±1.19	96.8±0.31	94.80±0.58	79.77±1.21	98.39±0.23
KPP (×100)	97.02±0.40	98.00±1.00	97.88±0.26	97.7±0.21	95.54±0.50	85.52±0.56	98.83±0.19

Bold values indicate the highest performance in each category.

**Table 6:** Individual class, OA, AA, and Kappa of all methods on the MUUFL dataset.

Class	MFT	ExVIT	Cross-HL	GAMF	HGCN-HL (HSI)	HGCN-HL (LiDAR)	HGCN-HL
1	97.41±0.51	98.56±0.11	98.13±0.30	98.07±0.31	98.41±0.06	94.98±0.47	98.61±0.07
2	91.67±1.74	89.71±1.13	88.74±1.74	87.39±1.10	92.67±0.67	61.41±6.35	94.16±0.67
3	89.79±1.58	90.73±0.96	90.66±1.74	88.54±1.10	91.24±0.76	67.75±5.01	92.27±0.54
4	91.69±3.06	94.24±1.30	94.74±1.55	94.52±1.50	93.79±0.50	57.75±6.26	92.80±0.64
5	94.62±1.36	95.84±0.58	95.81±0.59	95.15±0.79	96.65±0.36	77.26±2.50	96.86±0.14
6	76.70±3.57	88.22±2.82	87.20±5.75	92.42±1.49	92.91±1.25	93.84±1.78	94.97±0.95
7	87.34±3.64	93.97±1.10	92.27±1.14	92.22±1.97	93.04±0.51	52.41±4.91	92.94±0.56
8	96.52±0.75	97.74±0.12	98.15±0.33	98.03±0.45	97.72±0.25	95.38±0.90	98.08±0.16
9	49.68±7.65	68.17±2.41	67.13±3.15	65.85±2.99	66.13±1.84	7.63±2.52	71.70±1.47
10	1.15±1.00	32.30±4.71	23.91±7.75	20.75±7.12	17.64±3.38	0.00±0.00	28.28±4.44
11	68.40±5.22	77.41±5.40	70.51±3.26	68.28±1.94	71.48±2.90	0.27±0.82	75.27±4.37
OA%	93.03±0.21	94.92±0.11	94.49±0.18	93.97±0.06	95.06±0.17	80.56±0.79	95.65±0.10
AA%	76.82±1.28	84.50±0.78	82.48±1.09	81.93±0.97	82.88±0.50	55.33±1.24	85.09±0.42
KPP (×100)	90.76±0.29	93.29±0.16	92.71±0.25	92.03±0.18	93.46±0.22	74.07±1.02	94.24±0.13

Bold values indicate the highest performance in each category.

**Table 7:** The Overall Accuracy (OA), Average Accuracy (AA), and Kappa coefficient of different fusion methods on the three datasets.

Dataset	Metric	Add	Mul	Concat	Attention
Houston 2013	OA (%)	**91.31 ± 1.54**	89.92 ± 1.41	91.23 ± 1.29	90.07 ± 1.48
	AA (%)	**92.15 ± 1.29**	91.57 ± 1.14	92.12 ± 1.03	91.52 ± 1.09
	KPP (×100)	**90.56 ± 1.68**	89.05 ± 1.53	90.47 ± 1.41	89.22 ± 1.61
Trento	OA (%)	**99.13 ± 0.20**	97.93 ± 1.13	98.59 ± 1.07	98.57 ± 1.01
	AA (%)	**98.25 ± 0.60**	96.92 ± 2.56	97.93 ± 0.63	98.03 ± 0.67
	KPP (×100)	**98.83 ± 0.27**	97.24 ± 1.50	98.11 ± 1.41	98.09 ± 1.34
MUUFL	OA (%)	**95.65 ± 0.10**	95.28 ± 0.10	95.60 ± 0.09	95.36 ± 0.05
	AA (%)	85.09 ± 0.42	85.24 ± 0.78	**85.82 ± 0.48**	85.14 ± 0.68
	KPP (×100)	**94.24 ± 0.13**	93.75 ± 0.13	94.18 ± 0.12	93.87 ± 0.07

Bold values indicate the highest performance in each method.

**Table 8:** The Overall Accuracy (OA), Average Accuracy (AA), and Kappa coefficient of all methods on the Houston 2013 dataset under the limitedsamples.

Tr-Samples	3			5			7			9		
**Methods**	**OA**	**AA**	**KPP**	**OA**	**AA**	**KPP**	**OA**	**AA**	**KPP**	**OA**	**AA**	**KPP**
MFT	63.64	67.04	60.81	69.45	72.89	67.02	78.49	81.37	76.79	75.78	78.87	73.84
Cross-HL	19.32	18.80	13.30	21.84	22.34	16.17	28.88	29.47	23.60	28.56	30.79	23.47
ExVIT	64.07	69.73	61.35	78.69	80.93	77.01	86.88	88.10	85.82	85.00	86.42	83.79
GAMF	31.44	36.97	26.34	42.35	48.84	38.51	43.13	48.84	39.18	43.07	46.78	38.82
HGCN-HL	**70.60**	**75.03**	**68.23**	**81.62**	**82.19**	**80.12**	**85.78**	**87.16**	**84.61**	**88.52**	**89.36**	**87.58**

Bold values indicate the highest performance in each method.

**Table 9:** The Overall Accuracy (OA), Average Accuracy (AA), and Kappa coefficient of all methods on the Trento dataset under the limited samples.

Tr-Samples	3			5			7			9		
**Methods**	**OA**	**AA**	**KPP**	**OA**	**AA**	**KPP**	**OA**	**AA**	**KPP**	**OA**	**AA**	**KPP**
MFT	90.50	86.56	87.46	88.53	87.98	85.17	94.14	92.45	92.25	91.96	90.47	89.42
Cross-HL	76.22	56.75	66.43	77.13	62.41	67.99	79.9	76.12	72.04	79.83	76.26	72.05
ExVIT	92.17	86.55	89.49	94.66	92.56	92.93	94.53	91.74	92.76	98.82	96.42	98.43
GAMF	74.73	53.9	66.45	84.55	64.12	79.33	85.91	65.86	80.95	87.34	73.24	82.95
HGCN-HL	**94.95**	**87.82**	**93.22**	**96.56**	**95.86**	**95.45**	**97.82**	**96.02**	**97.09**	**99.30**	**98.52**	**99.06**

Bold values indicate the highest performance in each method.

**Table 10:** The Overall Accuracy (OA), Average Accuracy (AA), and Kappa coefficient of all methods on the MUUFL dataset under the limited samples.

Tr-Samples	3			5			7			9		
**Methods**	**OA**	**AA**	**KPP**	**OA**	**AA**	**KPP**	**OA**	**AA**	**KPP**	**OA**	**AA**	**KPP**
MFT	60.85	56.46	50.93	62.19	60.86	53.85	68.35	63.68	60.16	64.05	67.1	56.22
Cross-HL	20.43	29.18	12.71	22.91	31.21	14.54	30.18	35.87	20.87	35.55	41.57	26.35
ExVIT	69.15	65.84	61.12	68.69	65.06	60.49	78.86	75.04	72.75	78.29	73.84	72.21
GAMF	51.55	21.34	34.61	39.36	36.09	26.92	49.64	47.29	38.92	52.81	43.02	38.64
HGCN-HL	**76.43**	**67.60**	**69.69**	**78.76**	**76.72**	**72.74**	**85.52**	**81.58**	**81.10**	**84.69**	**82.47**	**80.32**

Bold values indicate the highest performance in each method.

**Table 11:** OA indices obtained from ablation experiments conducted on different datasets.

Dataset	WMF	CNNs	HGCNs	OA (%)
Houston 2013	✓	✓	✓	91.31±1.54
	×	✓	✓	89.23±0.75
	✓	×	✓	80.34±0.60
	✓	✓	×	90.52±1.12
Trento	✓	✓	✓	99.13±0.14
	×	✓	✓	97.51±1.17
	✓	×	✓	96.37±0.30
	✓	✓	×	94.79±2.78
MUUFL	✓	✓	✓	95.65±0.10
	×	✓	✓	92.27±0.17
	✓	×	✓	87.95±0.19
	✓	✓	×	95.02±0.10

**Table 12:** Running time(s) of different methods on each dataset.

Dataset	Time	MFT	ExViT	Cross-HL	GAMF	HGCN-HL
Houston 2013	Train(s)	270.4	565.07	203.71	2370.86	152.5
	Test(s)	0.27	1.7	0.39	7.96	2.38
Trento	Train(s)	157.67	314.14	123.43	3087.53	20.19
	Test(s)	0.23	3.55	0.53	16.29	0.17
MUUFL	Train(s)	135.79	715.11	249.82	5455.31	13.6
	Test(s)	0.41	6.03	0.81	27.24	0.13

## Data Availability

The Houston dataset used in this study is available at https://github.com/jingyao16/ExViT (accessed on 9 April 2025); the MUUFL dataset is available from https://github.com/GatorSense/MUUFLGulfport (accessed on 9 April 2025); the Trento dateset is provided by Lorenzo Bruzzone of the University of Trento and is available at https://github.com/giswl/HGCN-HL/tree/main/data/Trento (accessed on 9 April 2025).

## Abbreviations

The following abbreviations are used in this manuscript:

HSI	hyperspectral image
LiDAR	Light Detection And Ranging
HGCNs	hypergraph convolutional networks
CNNs	convolutional neural networks
